# 3D bioprinted CRC model brings to light the replication necessity of an oncolytic vaccinia virus encoding *FCU1* gene to exert an efficient anti-tumoral activity

**DOI:** 10.3389/fonc.2024.1384499

**Published:** 2024-07-18

**Authors:** Christophe A. Marquette, Emma Petiot, Anita Spindler, Caroline Ebel, Mael Nzepa, Baptiste Moreau, Philippe Erbs, Jean-Marc Balloul, Eric Quemeneur, Cécile Zaupa

**Affiliations:** ^1^ 3d.FAB, CNRS, INSA, Univ Lyon, CPE-Lyon, UMR5246, ICBMS, Université Lyon 1, Villeurbanne, France; ^2^ Transgene SA, Illkirch-Graffenstaden, France

**Keywords:** bioprinting, tumor, colorectal (colon) cancer, oncovirus, hydrogel

## Abstract

The oncolytic virus represents a promising therapeutic strategy involving the targeted replication of viruses to eliminate cancer cells, while preserving healthy ones. Despite ongoing clinical trials, this approach encounters significant challenges. This study delves into the interaction between an oncolytic virus and extracellular matrix mimics (ECM mimics). A three-dimensional colorectal cancer model, enriched with ECM mimics through bioprinting, was subjected to infection by an oncolytic virus derived from the vaccinia virus (oVV). The investigation revealed prolonged expression and sustained oVV production. However, the absence of a significant antitumor effect suggested that the virus’s progression toward non-infected tumoral clusters was hindered by the ECM mimics. Effective elimination of tumoral cells was achieved by introducing an oVV expressing FCU1 (an enzyme converting the prodrug 5-FC into the chemotherapeutic compound 5-FU) alongside 5-FC. Notably, this efficacy was absent when using a non-replicative vaccinia virus expressing FCU1. Our findings underscore then the crucial role of oVV proliferation in a complex ECM mimics. Its proliferation facilitates payload expression and generates a bystander effect to eradicate tumors. Additionally, this study emphasizes the utility of 3D bioprinting for assessing ECM mimics impact on oVV and demonstrates how enhancing oVV capabilities allows overcoming these barriers. This showcases the potential of 3D bioprinting technology in designing purpose-fit models for such investigations.

## Introduction

In the last decade, advancements in cancer treatment have significantly transformed the oncology landscape with the introduction of precision medicine and immunotherapies, demonstrating remarkable success in various cancer types such as lung cancers and melanoma (1, 2). These advancements were facilitated by early detection and improved characterization of the tumor microenvironment through genomics analysis and immune profiling, enabling tailored treatments. Despite these progressions, several limitations persist. Treatments for advanced-stage cancers remain restricted, and some cancers treated in the early stages develop resistance, evading treatments (3). Addressing such challenges remains a formidable task, possibly requiring the discovery of new therapeutic approaches or, more realistically, a combination of several treatment modalities. Therefore, gaining a deeper understanding of how tumor cells and their microenvironment respond to current treatments and assessing the obstacles they encounter would be highly beneficial.

With this objective in mind, the development and application of advanced tumor models have become indispensable. These models allow researchers to work with human cells and serve as valuable tools for unraveling the biology of tumors and understanding how therapeutics function. Over the past few decades, significant strides have been made in improving the replication of *in vivo* tumor environments, especially through the use of multicellular 3D models (4, 5). These models aim to mimic the interactions between various cell types, including tumor cells, fibroblasts, and endothelial cells (6). One significant benefit of *in vitro* models is the ability to recreate tumor environments with varying levels of complexity, permitting a step-by-step approach that facilitates a more comprehensive understanding of the role of different tumor components and their complex interplays (7). However, one critical aspect that is often overlooked is the presence of the extracellular matrix mimics (ECM mimics). The ECM mimics can profoundly affect the characteristics and progression of tumors while also acting as a barrier that hinders the accessibility of therapeutics. This issue becomes even more important when dealing with large molecules or biological agents such as antibodies or oncolytic viruses (OVs). Oncolytic viruses are designed to target and destroy tumor cells specifically, without harming healthy tissues. The viruses most commonly used in cancer virotherapy include vaccinia viruses, adenoviruses, herpes simplex viruses, and reoviruses (8). The originality of OVs lies in their multifaceted activities. They exhibit direct cytotoxic activity by engaging in the lytic virus cycle, resulting in the amplification of OVs specifically within the tumor site. Next, non-infected tumoral cells could be targeted with the expression of therapeutic genes by bystander effect, and finally, they could stimulate an immune cell response as foreign microorganisms (9). We have developed the oncolytic vaccinia virus TG6002, deleted of thymidine kinase and ribonucleotide reductase to enhance tumor-selective viral replication (10, 11). TG6002 expresses FCU1, a bifunctional chimeric protein that combines cytosine deaminase and uracil phosphoribosyltransferase activities and efficiently catalyzes the direct conversion of the non-cytotoxic prodrug 5-fluorocytosine (5-FC) into the chemotherapeutic compound 5-fluorouracil (5-FU) (12). TG6002 has recently entered clinical development in patients with advanced gastrointestinal tumors (NCT03724071, NCT04194034). These clinical trials are phase 1 studies evaluating the safety and tolerability of multiple-ascending doses of TG6002 administered intravenously in combination with oral 5-FC. Although several OVs have been evaluated in clinical trials in the last 20 years, only Talimogene laherparepvec (T-VEC) has been approved by the US Food and Drug Administration or the European Medicines Agency. T-Vec is used to treat patients with unresectable advanced melanoma recurrent after initial surgery (13, 14). Consequently, enhancing our knowledge of the mechanisms of action of OVs would help overcome potential obstacles and design the next generation of oncolytic viruses.

In this study, our primary goal was to design a model to assess how the extracellular matrix in the tumor microenvironment affects the efficacy of oncolytic viruses derived from the vaccinia virus (oVV). Here, we focused on the cytotoxic activity of oVV either directly through its replication or indirectly with the expression of an active payload in a matrix-rich tumoral model. Thus, we chose to create millimeter-scale 3D tumor models that incorporate authentic extracellular matrix components, allowing us to assess how this element of the tumor microenvironment affects its efficacy. Among the various techniques employed to create advanced models (15), we utilized 3D bioprinting technology, enabling the precise arrangement of cells and the extracellular matrix. We established 3D models consisting of colorectal tumor cells (HT29), cancer-associated fibroblasts (CAF), and a bio-inspired proliferative bioink (15). The bioink formulation (comprising gelatin, alginate, and fibrinogen) and its final mechanical properties (Young’s modulus) can be finely adjusted through the modulation of consolidation reactions (transglutaminase, calcium, and thrombin, respectively) (16). Once optimized and characterized, these models were used to evaluate the behavior of different oVVs in a complex 3D cellularized environment. We evaluated the expression, replication, and cytotoxicity of the oVVs in function of model stiffness. Taken together, our results show that the extracellular matrix affects oVV efficacy and that both virus replication and expression of a therapeutic payload are necessary to achieve antitumoral activity in a matrix-rich environment.

## Material and methods

### Virus

All oncolytic VACVs were derived from the Copenhagen strain and are deleted in thymidine kinase (*J2R*) and in the large subunit of ribonucleotide reductase (*I4L*) genes. oVV-GFP::FCU1 expressing an fusion protein between GFP and *FCU1* (*ΔI4LΔJ2R*/GFP::FCU1 VACV) and oVV-GFP expressing *GFP* (*ΔI4LΔJ2R*/GFP VACV) were constructed and characterized previously (10, 17). MVA-GFP::FCU1, the modified vaccinia virus Ankara (MVA) expressing fusion protein between GFP and FCU1, was constructed and characterized as described previously (18). MVA-FCU1::GFP and recombinant VACVs were amplified in primary chicken embryonic fibroblasts (CEFs) and purified. MVA-FCU1 and oVVs virus stock were titrated by plaque assay on CEFs and Vero cells respectively.

### Tumor model production

Tumor models were produced through 3D bioprinting (19). 3D bioprinting bioinks was formulated using bovine gelatin (Merck #G1890), very low viscosity alginate (Alpha Aesar, # A18565) and fibrinogen (Merck, # F8630), each dissolved overnight in DMEM without calcium (Gibco™ #21068028) at 37°C. Stock solutions of 0.2 g/mL gelatin, 0.04 g/mL alginate and 0.08 g/mL fibrinogen were prepared in Dulbecco’s modified eagle’s medium (DMEM without calcium (Gibco #21068028) with glutamax-1, Gibco #35050061) supplemented with 10% Fetal Bovine Serum (Thermofisher scientific #A3160802), 100 UI/ml Penicillin-Streptomycin (Thermofisher Scientific, #15140122) and 1 µg/ml amphotericin B (Thermofisher scientific, #15290026).

Then, just before bioprinting, the bioink was formulated as 0.02 g/mL of fibrinogen, 0.02 g/mL of alginate and 0.05 g/mL of gelatin, and seed with cells. To do so, cancer associated fibrobasts (Neuromics, #CAF05) and colorectal cancer cells (ATCC, HT29) were trypsinized and resuspended in 0.08 g/mL fibrinogen at a concentration of 1.5 10^6^ cell/ml and 5 10^6^ cell/ml, respectively. To 2mL of this suspended cell solution, 4 mL of alginate stock solution and 2 mL of gelatin stock solution. After homogenization, a 10 mL sterile cartridge (Nordson EFD, France) was filled with the bioink, incubated 15 minutes at 37°C and then 30 minutes at room temperature (21°C) to stabilize the bioink rheological properties. The cartridge was then loaded in a 6-axis robotic bioprinter (BioAssemblyBot^®^, Advanced Lifescience Solutions) and used to print standardized 0.3 cm3 bioprinted tissues (1cm*1cm*2mm). A 410 µm diameter, 6.35 mm long needle (Nordson EFD) was used to bioprint at a set speed of 10 mm/sec.

Once bioprinted, the tumor models were consolidated for 90 minutes at 37°C in a solution containing the following components: 270 mM of CaCl_2_ (Sigma, France), 40 or 4 mg/mL of transglutaminase (Ajinomoto, #ActivaWM) and 10 U/mL of thrombin (Sigma, #T4648). Once the consolidation completed, each bioprinted tissue was rinsed three times with sterile NaCl 0.9% (Versol, #69600501).

The produced tumor models were then cultured in 12-well plates containing 2 mL of RPMI (ATCC, #30–2001) supplemented with 10% FBS, at 37°C in a 5% CO_2_ incubator.

More than 300 models were produced during this study.

### DMA measurements

The viscoelastic behavior of the tumor model was characterized by frequency sweep experiments in dynamic mechanical analysis (DMA) in compression mode. These experiments were conducted with a rotational rheometer (DHR2, TA Instruments, Guyancourt, France) with a DMA mode (torque = 0N) using disk-shaped samples and a parallel plate geometry (8 mm). A preliminary study was performed to define the linear viscoelastic domain, which corresponds to the displacement range where the material properties are assumed to be constant. This domain is determined using oscillatory compression experiments with constant frequency and varying displacement. Then, dynamic compression tests were performed with a frequency range of 0.1 to 10Hz (*i.e.* 0.628 to 62.8 rad/s) at a constant displacement, which is within the linear viscoelastic regime. In these dynamic compression tests, tumor model undergoes a periodical mechanical strain ϵ of very small amplitude $\epsilon_{0}$ and of angular frequency ω following the Equation 1:


(1) $$\varepsilon =\;\varepsilon_{0}\;sin\;sin\;\left(\omega t\right)\;$$

In the case of the generalized Maxwell model, the storage E′(ω) part of the complex modulus is expressed by the Equation 2:


(2) $$E^{\prime }\left(\omega \right)=\;E_{0}\left[1+\sum_{\alpha =1}^{m}\;\frac{\beta_{\alpha }\omega^{2}\tau_{\alpha }^{2}}{1+\omega^{2}\tau_{\alpha }^{2}}\right]$$

(3) $$\eta =E_{0}\sum_{\alpha =1}^{m}\;\beta_{\alpha }\tau_{\alpha }$$

where $E_{0}$ is Young’s modulus of the isolated spring. The relaxation times, ${\;}_{\tau }^{\alpha }$ , and the dimensionless reference parameters $\beta_{\alpha }$ stand for the contribution of each branch to the global modulus. The overall viscosity η can be defined as Equation 3. The time-constant values were regularly distributed between the reciprocals of the highest (62.8 rad/s) and the lowest (0.628 rad/s) angular frequencies of the experimental dynamic modulus. The chosen number of modes was sufficiently high to obtain accurate fitting, but not too large to avoid inconsistent results (*e.g.*, negative values of $\beta_{\alpha }$ ). Practically speaking, this led to three-time constants (m = 2), regularly spaced on a logarithmic scale between 5 × 10^−2^ s and 5 × 10^−1^ s.

Identification was achieved by solving the following minimization problem described by Equation 3:


(3) $$f_{obj}\left(E_{0},\beta_{1}\ldots \beta_{m}\right)=\sum_{i}^{k}\;\left[\frac{{\left(E_{\;i}^{\prime }-E_{\;i}^{\prime mes}\right)}^{2}}{E_{\;i}^{\prime mes}}\right]$$

where $E_{i}^{'mes}$ is the storage modulus obtained from the measured data and $E_{{\;}_{i}}^{'}$ is the one computed with viscoelastic parameters. k is the number of measurements acquired during the frequency sweep compression test. The optimization procedure was performed by using the Microsoft Excel Solver (version 2016) with the Generalized Reduced Gradient (GRG) nonlinear solving method.

### Macroscopic analysis

Macroscopic follow up of 3D model growth and viability was performed on a stereo fluorescence microscope (Nikon SMZ18) by transmitted light and green fluorescence following staining of viable cells with Calcein cell-permeant dye at 1 µg/mL (Invitrogen™, #C3100MP).

### Histological and immunofluorescence characterization

Histological assessment was made using paraffin-embedded 3D bioprints after fixation in 4% formaldehyde-16.7mM CaCl_2_ solution. Paraffin embedded 3D bioprints were then cut in 4µM sections and mounted on slides. Sections were deparaffinized, hydrated, and then hematoxylin and eosin (H&E) and Masson-Goldner trichrome (Bio-optical) staining were performed for pathomorphological assessment. Immunohistological analyses for Ki-67, EpCam and Cleaved-caspase 3 were carried out on Bond RXM (Leica). After deparaffinization and rehydration, epitope retrieval was performed using Bond epitope retrieval solution 2 (Leica, #AR9640). Endogenous peroxidase activities were blocked by incubating sections in Hydrogen peroxide block solution (Labvision, #TA-125-H2O2Q), for 10 minutes at room temperature. Sections were saturated with 10% goat serum for 20 minutes. Primary rabbit antibodies rabbit anti-Ki67 (LS-Bio, #LS-B13463), mouse anti-EpCam (Cell Signaling technology, #2929), and rabbit anti cleaved-caspase 3 (Cell Signaling Technology, #9661) were incubated 45 min at room temperature. The mouse anti-Epcam antibody was revealed using Post Primary (rabbit anti mouse secondary antibody Leica, #RE7159), followed by Novolink anti-Rabbit polymer (Leica, #RE7161), the two other antibodies were revealed using Novolink anti-rabbit polymer alone. Finally, a Tyramide System Amplification step (TSA-Fluorescein, # SAT701001EA) and a counter staining step using bis-Benzimide Hoechst3328 (Sigma-Aldrich, #B-2883) were realized.

Hypoxia areas of 3D tumor models were investigated using labeling of pimonidazole hydrochloride (PIMO) which forms stable covalent adducts with thiol in hypoxia. The staining was performed using Hydroxyprobe Omni Kit (Hydroxyprobe, #HP1–100Kit) according to the manufacturer’s protocol. Briefly, models were incubated for 3hrs with a growth medium supplemented with 200 µM of PIMO, washed two times with PBS and fixed overnight with 4% formaldehyde and Cacl2 (16.7 mM). Next, models were dehydrated, paraffin embedded and cut in 4µm sections. The slides were treated as described previously with the Bond RXM except for the epitope retrieval which was performed with Bond epitope retrieval solution 1, and the primary antibody which was a rabbit IgG anti-Pimonidazole (1/1000; PAb2627, Hydroxyprobe).

### Image analysis

ImageJ was used for image analysis and cluster quantification. Briefly, full color microscopy images were transformed into 8-bit black and white images and saved. A pixel intensity threshold was then applied to the transformed image and the function “Analyze Particles” applied to segment all clusters. Clusters were then sorted out according to their position in the initial image to plot their distribution within the bioprinted tumor model.

### Infection

Oncolytic virus infection was conducted with 3D models cultivated within 6-well plate. The 3D models were infected 40 days post-printing when cell clusters were fully established. The culture medium was reduced to a volume of 3 mL ensuring it just covered the surface of the 3D models. Next, a defined amount of oVV was carefully administered drop by drop on the top of each model. The infected 3D tumor models were then incubated under controlled conditions at 37°C with 5% CO2. Following 3 hours, the culture medium was replenished to a final volume of 5mL and finally the models were maintained at 37°C with 5% calf serum during. Culture medium with 5% calf serum was changed every 2 or 3 days to ensure optimal growth conditions. When models were infected with oVV-GFP, infection of 3D tumor models was monitored by following GFP expression from the oVV-GFP with a stereo fluorescence microscope. Models were incubated at 37°C and 5% CO2 during the entirety of the experiment.

### Viability assessment

The viability of the 3D bioprinted tumor models was assessed by measuring the metabolic activity of the cells using resazurin reduction using CellTiter-Blue assays (Promega, #G8080) Briefly, at the indicated time, the 3D models were transferred in RPMI supplemented with 10% FBS and 20% CellTiter-Blue. After 9 hours of incubation, 100uL of medium was transferred to 96 wells and the reduction of resazurin was determined (560/590 nm excitation/emission filters) with a Tecan Elisa Reader. Signal of infected 3D models were compared to control models treated with 10% RPMI medium to calculate the percent of viability.

### Enzymatic activity assessment

Evaluation of cytosine deaminase activity was quantified by measuring the amount of 5-FU released in the culture media. After 4 days of infection by oVV-GFP::FCU1 at 10^5 and 10^6 PFUs, 1 mM 5-FC (Toronto Research Chemicals Inc., #F589000) was added to the culture medium. Every 2/3 days, medium was renewed with 5-FC at 1 mM and 5-FC and 5-FU concentrations in the media were measured by high-performance liquid chromatography. Twenty microliters of media were analyzed by high-performance liquid chromatography using a mobile phase of 50 mM phosphoric acid adjusted to a pH of 2.1. Results are expressed as the percentage of 5-FU relative to the total amount of 5-FC plus 5-FU.

## Results

### 3D bioprinting allows the generation of tumor models with an environment rich in extracellular matrix

The primary objective of this study is to explore novel therapeutic approaches within a realistic tumor environment. This involves the creation of human cell-based models that incorporate extracellular matrix as it plays a crucial role in cell metabolism and the effectiveness of anti-tumor treatments.

*In vitro* models of colorectal cancer (CRC) were meticulously constructed using 3D bioprinting, employing HT29 colorectal tumor cells along with colorectal cancer-associated fibroblasts (CAF) to emulate a representative CRC environment. These co-cultured cells were precisely layered within a proprietary bioink that supports cell growth and biocompatibility (15, 20). The stiffness of these initial models was characterized by a 12 kPa Young’s modulus (obtained through dynamic mechanical analysis). The models were cultivated under controlled conditions for over 35 days and extensively characterized. Live cell growth, structure, and organization were visualized through calcein staining, while immunohistochemistry was utilized to identify markers for cell proliferation, apoptosis, hypoxia, and cancer phenotypes.

**Figure 1:**
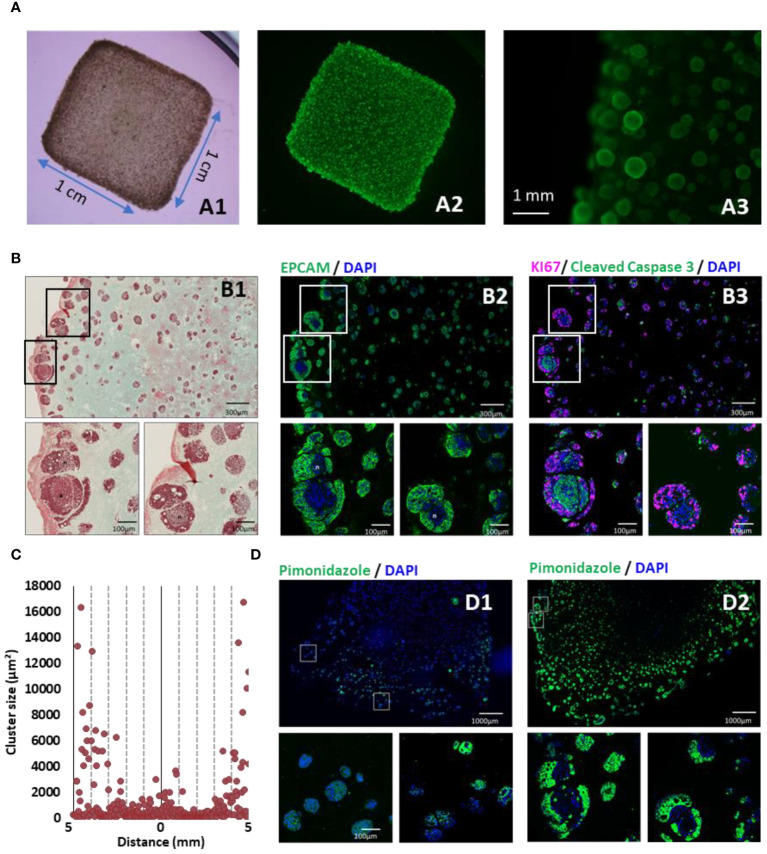
3D-bioprinted tumor models characterization. **(A)** 35 days after 3D printing, models were stained with calcein AM and were observed in bright field **(A1)**, under fluorescent light to detect live cells **(A2)** and at higher magnification **(A3)**. **(B)** Models were fixed, paraffin embedded and sliced. Consecutive sections were analyzed by Masson’s trichrome staining **(B1)**, EPCAM (green) **(B2)**, and immunofluorescent staining against KI67 (purple) and cleaved caspase 3 (green) **(B3)**. **(C)** Cluster size distribution along 10 mm CRC model histological sections, quantified using imageJ software and particle analysis. **(D)** Hypoxia was monitored by short incubation with pimonidazole followed by specific immunofluorescent staining at 10 days **(D1)** or 35 days **(D2)** post-printing. Nucleus were counter-stained with DAPI (Blue).

After 35 days of culture, viable cellular spheroids, labeled with calcein, were evident in the CRC model (**Figure 1A**). However, an analysis of the 3D bioprinted models, aided by histochemistry, revealed an uneven distribution of cell clusters within the hydrogel. Larger and more numerous cell clusters were observed at the periphery, particularly within the first 1000 µm near the edge of the structure (**Figures 1B1, C**). This distribution of cell clusters is consistent with prior findings for similar bioprinted structures and is associated with a gradual reduction in nutrient and oxygen availability (21). The presence of the EPCAM glycoprotein (**Figure 1B2**), an adenocarcinoma marker, confirmed that these cell clusters primarily consisted of HT29 cells. Masson’s trichrome green staining suggests that the surrounding extracellular matrix, is composed of collagen. Based on control experiment with gelatin hydrogel in absence of cell (see **Supplementary Figure 1**), and on literature it is hypothesized that this collagen was produced by cancer-associated fibroblasts during the initial 35 days of culture (22, 23). Further investigations are needed to confirm it.

Proliferation and apoptosis in the cell clusters was also assessed using Ki67 and Caspase 3 labeling (**Figure 1B3**). Interestingly, hypoxia, monitored through pimonidazole labeling, did not vary significantly based on the location within the model but was associated with the time elapsed post-printing and the growth of the clusters (**Figure 1D**). Notably, at 35 days post-printing, cell clusters exhibited distinct regions with actively proliferating cells at the outer layer and apoptotic cells at the center of the cluster (**Figure 1B3**). The hypoxic region was located in the intermediate area where cells were actively proliferating.

In summary, the 3D bioprinted CRC models successfully replicated critical characteristics of tumors, including heterogeneity in cell proliferation, hypoxia, and the presence of a dense extracellular matrix.

### Oncolytic virus infects and maintains long term expression in 3D bioprinted CRC models

The 3D CRC models were created to facilitate the study and development of virus-mediated cancer treatments, with a particular focus on understanding the impact of the ECM mimics. In the initial phase, we evaluated the influence of ECM mimics on the replication and direct killing of oncolytic vaccinia virus (oVV). To achieve this, we employed a first-generation GFP-expressing oVV (oVV-GFP), which is engineered to enhance tumor-selective viral replication by deleting thymidine kinase and ribonucleotide reductase. Additionally, it expresses the GFP protein as a fluorescent marker to monitor virus propagation and expression within the complex bioprinted environment.

**Figure 2:**
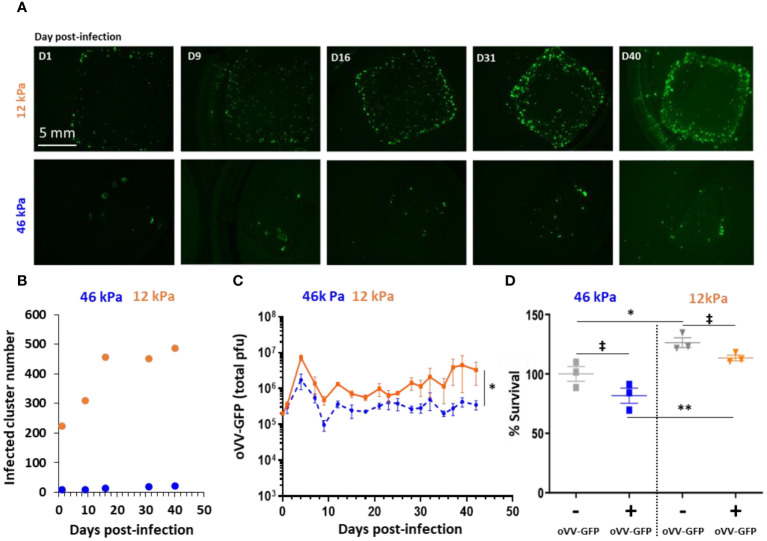
Stiffness of 3D bioprinted tumor model impacts infection and proliferation of oncolytic Vaccinia Virus. 3D bioprinted tumor models with different matrix stiffness were infected 40 days post-printing by 10^5 PFU of oVV expressing eGFP. **(A)** Virus expression was monitored by fluorescent microscopy during at least 40 day post-infection. Matrix with stiffness of 46 kPa (top) or 12 kPa (bottom) were assessed. **(B)** Numbers of infected-clusters by oVV-GFP were quantified using imageJ software and particle analysis. **(C)** Following infection, supernatant was renewed every 2–3 days and oVV presents in the supernatant was titrated. The orange line and the dashed blue line represented the mean of the total PFU ± SEM (n=3) for the model with 12 kPa and 46 kPa stiffness respectively. **(D)** At 42 days post-infection, the viability of the models was determined using Celltiter Blue assay. The 100% viability was based on mock-treated models. Symbols represent individual models, and horizontal lines indicate the mean ± SEM (n=3). ‡: non-significant ANOVA test (p>0.1). *: significant ANOVA test (p<0.02). **: significant ANOVA test (p<0.002).

Regarding the mechanical properties of the 3D CRC models, the initial Young’s modulus, representing the stiffness of the material, was measured at 12 kPa. This value falls a little higher than the range defined for healthy colorectal extracellular matrix (1 to 5 kPa) and is slightly below the values for CRC extracellular matrix (25 to 50 kPa) (24). To investigate the impact of stiffness on oVV, two CRC model populations with different stiffness levels were generated. This was achieved by adjusting the gelatin reticulation within the bioink through varying transglutaminase concentrations during model consolidation (4 or 40 mg/mL), resulting in final stiffness values of 12 and 46 kPa. Both models were infected with oVV-GFP at 40 days post-printing, and the infection and virus propagation were monitored through fluorescent microscopy (**Figure 2A**).

In the 46 kPa CRC models, oVV-GFP successfully infected cell clusters, with GFP clusters observed as early as day 1 post-infection. However, the number of infected clusters at day 1 was minimal (8 clusters), and this number did not exceed 21 even at day 40 post-infection (**Figure 2B**). These results suggest that oVV-GFP continued to be expressed at day 40, although without clear virus diffusion within the model.

In the 12 kPa CRC models, GFP clusters were also observed at day 1 post-infection, and their numbers (224 clusters) were higher than in the 46 kPa CRC. These numbers continued to increase until day 16, and then in the following days, the number of infected clusters remained stable (around 450). At 40 days post-infection, GFP expression (indicative of virus expression) was more pronounced and higher compared to the 46 kPa models. In parallel, analysis by immunostaining of slices derived from 12 kPa models after 18 days post-infection showed oVV-GFP infection in only a portion of the clusters (**Supplementary Figure 2**).

The amount of virus released into the culture medium of the two CRC model populations supported these findings (**Figure 2C**). Significantly more virus was released from the 12 kPa models compared to the 46 kPa models, aligning with the observed GFP expression within the models. The kinetics of oVV release were similar in both models, with an initial burst of oVV at day 6 post-infection, followed by a decline in oVV titers. Subsequently, the release of oVV reached a plateau, which was maintained for at least 42 days post-infection when the culture was concluded. Notably, the plateau of virus production was lower in the stiffer 46 kPa models, suggesting that virus proliferation and propagation were hindered by the degree of extracellular matrix reticulation.

Lastly, cell viability was assessed by measuring their metabolic activity, with 100% viability set for the 46 kPa non-infected model (**Figure 2D**). Regardless of infection, it was observed that the cells in the 12 kPa models exhibited higher metabolic activity. Surprisingly, there was no significant difference (ANOVA statistical test) in viability between the infected and non-infected models, irrespective of the model’s stiffness. This indicates that virus infection did not impact tumor viability, despite the continued presence and expression of the virus even 42 days post-infection. Conversely, in a 2D environment, oVV is able to replicate in HT29 and efficiently kills cells with a viability less than 5% at an MOI of 0.01 five days post infection (**Supplementary Figure 3**). Possible explanations for the limited tumor cell killing in the 3D CRC models include the virus’s restricted ability to propagate through the extracellular matrix or the resistance of tumor cells in a 3D configuration.

### Expression of payload increased the antitumor efficiency of second generation oVV

The first-generation oncolytic vaccinia virus (oVV) showed limited impact on the viability of HT29 cells within the 3D models, primarily due to challenges in propagating through the bioprinted extracellular matrix. Recognizing the limitations of direct lysis activity, a strategy involving a second-generation oVV expressing a therapeutic gene was explored to enhance therapeutic effectiveness. In this study, the tested oVV expresses an enzyme called FCU1 fused with GFP (oVV-GFP::FCU1). FCU1 has the ability to convert a non-toxic prodrug, 5-fluorocytosine (5-FC), into the active chemotherapeutic agent 5-fluorouracil (5-FU), with GFP facilitating direct fluorescence monitoring of virus expression.

**Figure 3:**
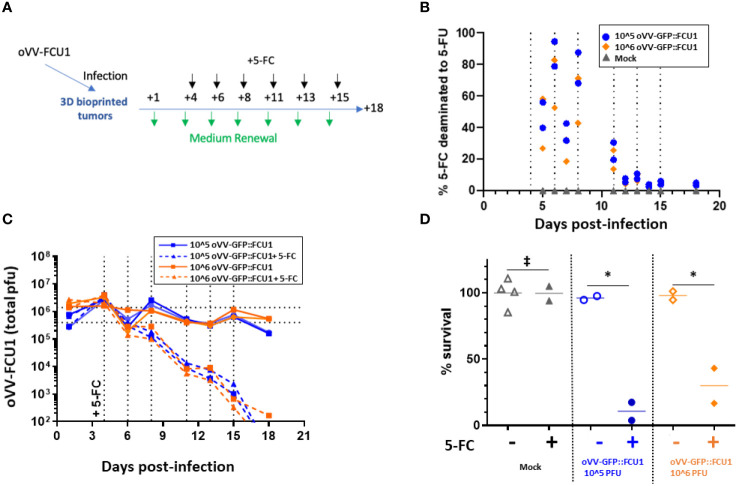
oVV expressing GFP::FCU1 as payload demonstrates efficient antitumor cytotoxicity. **(A)** Schematic depicting the experimental procedure to evaluate the antitumor efficiency of oVV-GFP::FCU1. 3D bioprinted-tumor models were infected with 10^6 or 10^5 PFU of oVV-GFP::FCU1, the following day input virus was removed and new medium added. Then, 4 days post infection 5-FC (1mM) was added in new medium. Every 2/3 days medium with 5-FC was renewed. Experiment was stopped 18 days post-infection and model viability was measured. As control, same procedure was applied with medium without 5-FC to infected and non-infected models (for each condition with infected models (n=2), mock models + 5-FC (n=2) and mock models (n=4). **(B)** Concentration of 5-FC and 5-FU in model supernatants was determined by high-performance liquid chromatography. The results are presented as percent of 5-FU generated from 5-FC with each symbol representing an individual model The percent of 5-FC/5-FU conversion was defined in medium before the medium was renew. **(C)** Virus in the supernatant was quantified by PFU titration. Each line shows the total PFU measured for individual models. Dotted vertical line indicated the addition of new medium with 5-FC **(D)** Viability of the models was determined at 18 days post-infection using Celltiter Blue assay. The 100% viability was based on mock-treated models. Symbols represent individual models, and the horizontal line indicates the mean. ‡: non-significant ANOVA test (p>0.9). *: significant ANOVA test (p<0.02).

In the present study, 46 kPa CRC models were infected with different quantities of oVV-GFP::FCU1 (10^6 or 10^5 plaque-forming units) and then cultured for four days. Subsequently, the models were treated with 1mM of prodrug 5-FC every 2–3 days (**Figure 3A**). Regardless of the initial virus quantity used during infection, 5-FC was efficiently converted into 5-FU, with 100% conversion by day 5, showcasing the effective expression and cytosine activity of FCU1 (**Figure 3B**). However, after day 5, the enzymatic activity began to decline, reaching a negligible value of less than 5% by day 12 post-infection. This reduction in enzymatic activity corresponded to a decrease in virus release at day 8 post-infection compared to models infected in the absence of 5-FC (**Figure 3C**). Ultimately, the infection by oVV-GFP::FCU1 with the addition of 5-FC induced a significant antitumor effect, with a reduction in viability to less than 40% at 18 days post-infection and 14 days post addition of 5-FC for both virus doses (**Figure 3D**). As expected, neither oVV-GFP::FCU1 nor 5-FC alone had any effect on the viability of the 3D bioprinted models even with a higher dose of 10^6 PFU. In summary, these findings demonstrate that following infection, oVV-GFP::FCU1 efficiently expresses the FCU1 enzyme, leading to the conversion of 5-FC to 5-FU, resulting in the death of the 3D bioprinted tumor models. This decrease in tumor cell content within the models subsequently led to a reduction in virus proliferation and expression, as live proliferating cells are required for these processes.

### Virus proliferation is essential to the antitumor activity

Given the absence of any antitumor activity observed with oVV alone, the study sought to determine whether a replicating virus was essential or if the activity of FCU1 alone is responsible for the antitumoral response. To address this, the study compared the antitumoral effects of VV-GFP::FCU1 and MVA-GFP::FCU1. MVA-GFP::FCU1 is an attenuated vaccinia virus that cannot replicate in human cells but encodes the GFP::FCU1 gene under a similar promoter as VV-GFP::FCU1. Both viruses were applied to 3D bioprinted tumor models at 10^5 PFU, following **Figure 3A** schedule.

**Figure 4:**
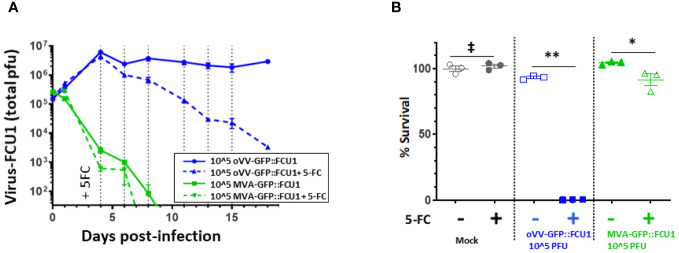
oVV-GFP::FCU1 replication is needed for efficient antitumoral and bystander effect. **(A)** 3D bioprinted-tumor models were infected by 10^5 PFU of oVV-GFP::FCU1 or MVA-GFP::FCU1 as described in **Figure 3A**. 5-FC (1mM) was added 4 days post infection and every 2/3 days medium was renewed with 5-FC. Virus in the supernatant was quantified by PFU titration. Results are represented as the mean of the total PFU ± SEM (n=3). As a control, identical protocol was carried over without the addition of 5-FC. Dotted vertical line indicated the addition of new medium with 5-FC **(B)** 18 post-infection the viability of the models was determined using Celltiter Blue assay. The 100% viability was based on mock-treated models. Symbols represent individual model, and horizontal line indicates the mean ± SEM (n=3). ‡: non-significant ANOVA test (p>0.9). **: significant ANOVA test (p<0.002). *: significant ANOVA test (p<0.02).

As anticipated, MVA-GFP::FCU1 was incapable of replicating in both conditions, with or without 5-FC. On the other hand, the replication of VV-GFP::FCU1 remained constant without 5-FC, but, as previously observed (**Figure 3C**), it decreased after the addition of 5-FC (**Figure 4A**). Intriguingly, significant but moderate antitumor activity (85% cell survival) was observed with MVA-GFP::FCU1 in the presence of 5-FC, whereas highly efficient antitumor activity (100% cell death) was observed with the combination of oVV-GFP::FCU1 and 5-FC (**Figure 4B**). These results unequivocally demonstrate that, while the replication of oVV-GFP::FCU1 alone may not be sufficient to induce antitumor activity, it is indeed necessary. This study suggests that in an environment where optimal oVV proliferation is hindered, such as by the extracellular matrix or resistant cells, even minimal virus proliferation can still play a role by maintaining long-term expression of a therapeutic payload.

## Discussion

In this study, we designed a new 3D bioprinted CRC model to investigate the impact of the ECM mimics and its stiffness on oVV efficiency. This seemingly straightforward question has proven challenging to address with smaller models, such as multicellular spheroids, where ECM is limited, or *in vivo*, where regulating and measuring ECM composition and stiffness are formidable tasks.

Several studies have been conducted to develop more predictive models and to maximize the efficacy potential of oncolytic viruses. Initial efforts involved the use of spheroid tumor models, which recapitulate heterogeneous metabolism of tumor cells based on their spatial location within the tumor. This includes proliferative cells on the periphery and more quiescent and necrotic cells toward the core. Studies employing various oncolytic viruses have demonstrated reduced efficacy in such 3D models, suggesting potential limitations related to slower cell metabolism or restricted virus spreading within the 3D environment (25, 26). Then, the generation of organoid models derived from patient tissues, allows for the exploration of patient variability and the heterogeneous cellular nature of tumors. Oncolytic viruses derived from Measles and Vaccinia Virus, tested in patient-derived organoids of primary breast cancer, exhibited minimal patient-to-patient variability but efficiency in a three-dimensional configuration was enhanced by payloads expression (27). Conversely, the use of patient-derived pancreatic tumor organoids to screen oncolytic adenoviruses highlighted the response variability among different patients (28). These investigations underscore the value of three-dimensional models in gaining understanding of oncolytic virus mechanisms and in developing more pertinent payloads. Furthermore, these studies hold promise for personalized treatments, with screening of an oncolytic virus bank. However, the ECM composition in these models is often limited to Collagen or Matrigel, which have significantly lower stiffness than *in vivo* tumors (29). Organotypic slices of tumor tissues or tissue explants represent an alternative approach, as they retain the original components and organization of the tumor. These have been leveraged for evaluating the efficacy and safety of oncolytic viruses (30, 31). Nevertheless, maintaining their viability poses a significant challenge, necessitating efficient handling and coordination between the operating room and the research laboratory with a viability often limited to one week.

Moving beyond 3D models described above, our study harnessed the innovative potential of 3D bioprinting to address the shortcomings in ECM complexity and long-term viability to explore the impact of the ECM mimics and its stiffness on oVV effectiveness. The utilization of 3D bioprinting technology allowed us to exert control over the models’ size (reproducible shape and dimension), composition (bioink formulation), and, most importantly, their mechanical properties through the fine-tuning of the bioink reticulation. Indeed, the stiffness of ECM in cancer is often higher than in normal tissues and further increases as the disease progresses (32). The bioink composition was formulated to mimic as much as possible the typical composition of mammalian soft tissues, i.e. 60–65% water, 16% protein (comprising collagen and other extracellular matrix components), 1% carbohydrate (33).

Two levels of stiffness were implemented in the generated models: the lowest at 12 kPa, higher than that of healthy colorectal ECM but lower than in CRC, and the highest at 46 kPa, falling within the range of CRC Young’s modulus. Comparing oVV under these two conditions revealed that stiffness influences oVV progression. The impact can be direct, as the higher reticulation of the matrix can physically impendes oVV. Alternatively, it can be indirect, affecting cell metabolism. We showed that the metabolic activity in the 46 kPa model is lower than the 12kPa model. Given that oVV-GFP lacks TK and RR viral enzymes, its replication relies on cellular enzymes. Consequently, lower cell metabolism in the 46 kPa models may limit oVV replication. It is noteworthy that, even in 12 kPa model virus, progression and antitumoral action were limited. Previous study has demonstrated that ECM rich in lamin may impaired spreading of oncolytic virus derived from HSV-1 to infect cells and also its replication after the entry (34). Our study is to our knowledge the first to strongly suggest that beyond the ECM composition, its stiffness impedes the virus propagation.

Further exploration may involve varying the Young’s modulus to determine if a reduction enables enhanced oVV propagation among other clusters, or if its propagation limitation is mainly due to ECM mimics volume between tumoral clusters. This question may then be addressed with higher cell density or different architecture that can be easily created through bioprinting. The models generated here may serve to assess strategies allowing oVV to counteract the ECM mimics, either by expressing degrading enzymes or by combination with therapies that soften it. However, such strategies are complex, as non-selective ECM mimics degradation could lead to increased tumor growth or metastasis (35). Hence, easy screening models, as the one described here, will be a valuable asset. On another note, it would be very tempting to complexify this model to incorporate additional types of cells, like immune cells, which are not only affected by interaction with tumor cells but also with ECM mimics, and to study their behavior or their impact on oVV therapies.

The ability to maintain the 3D bioprinted model in culture for over 2 months is of significant value; it allows us to follow oVV infection for an extended period, which is not possible in classical 2D models or even in multicellular spheroids. This led to the surprising observation that virus production continued for more than 45 days without affecting tumoral viability. The lack of impact on tumor viability could be rationalized by the small number of clusters, and thus cells, that are reached by oVV due to ECM mimics, as explained previously (< 5% infected cells at d+5 and d+28 post-infection, **Supplementary Figure 4**). However, the continuous virus release and expression are intriguing, considering the VV viral cycle in 2D cell culture is less than 12 hours and results in the killing of infected cells (36). Consequently, it would be expected that after a few days, the virus will propagate from cell to cell in the infected cluster to induce its destruction and then be stopped due to the absence of living cells and the impossibility to reach new cell clusters due to ECM mimics. One hypothesis could be that cells are proliferating at a similar rate than the virus cycle, thus leading to the replacement of killed cells and sustaining new oVV production. Another hypothesis could be that in 3D bioprinted models, the oVV cycle is modified. It may be longer, not taking just several hours but several days. It has been well-documented that tumor cell expression and properties are modified when in 3D cultures compared to 2D. For example, such changes render them more resistant to treatment (37–39). They may become more resistant to cell death induced by oVV and may favor the production of extracellular enveloped virus. Indeed, two kinds of infectious vaccinia virus particles are typically produced during the viral cycle: the intracellular mature virus (IMV) and the extracellular enveloped virus (EEV). The IMV is released upon cell lysis, while EEV release results from exocytosis or budding through the plasma membrane (40). Characterizing the oVV particles (IMV vs EEV) and comparing cell expression in a 2D versus 3D bioprinted configuration may provide valuable insights to understand our results.

Finally, we demonstrated that if a first-generation oVV lacks effectiveness by itself, the induction of a bystander effect through the expression of a therapeutic gene, such as FCU1, will destroy the tumor cells. No dose effect was observed in 5-FC to 5-FU conversion, virus release, or cell viability. While surprising, this may indicate that a limited number of cells can be reached independently to the dose and that payload expression in those cells is sufficient. Exploring the efficacy of lower virus dose combined with 5-FC will be interesting. However, using a non-replicating vector derived from the vaccinia virus at the same dose, we show that expression of FCU1 with the addition of 5-FC was insufficient for eliminating tumor cells. If the bystander effect of FCU1 due to the conversion of produg 5-FC into the potent chemotherapeutic agent 5-FU has been previously demonstrated_ENREF_11 (12), the necessity of virus replication has not been proven before. It highlights the added value of a replicating virus in comparison to an expression vector, despite conditions in which replication and propagation of the virus are diminished by a complex environment. Moreover, given that oVV is capable of maintaining a long-term expression of the transgene, this is highly encouraging for clinical application, especially in scenarios where viruses may realistically not attain every tumor cell, and the bystander effect is anticipated to play a crucial role in therapeutic activity.

### Conclusion and future outlook

In this study, we successfully established a 3D tumor model rich in ECM mimics, enabling the investigation of obstacles encountered by oncolytic vaccinia viruses (oVVs) during their application. We demonstrated that ECM mimics limits their propagation and direct cytotoxic activity. However, the addition of therapeutic payloads like FCU1, in combination with 5-FC, allows the targeting of additional tumoral cells and efficient antitumor activity. Importantly, to achieve this efficacy, oVV should maintain some level of replication. These results underscore the complementary roles of virus replication and payload expression, emphasizing the essential nature of multiple therapeutic modalities for effective treatment.

Moreover, this study serves as a proof of concept, showcasing how the flexibility of bioprinted 3D models can help address specific questions regarding the mode of action of treatments. The further incorporation of immune cells into this 3D bioprinted model offers an interesting avenue to tackle questions about the interplay of oVVs, tumor cells, and immunity, with the aim of leveraging immune cell properties to destroy the tumor without hindering oVVs.

## Data availability statement

The raw data supporting the conclusions of this article will be made available by the authors, without undue reservation.

## Ethics statement

Ethical approval was not required for the studies on animals in accordance with the local legislation and institutional requirements because only commercially available established cell lines were used.

## Author contributions

CM: Conceptualization, Data curation, Formal analysis, Funding acquisition, Investigation, Methodology, Project administration, Validation, Writing – original draft, Writing – review & editing. EP: Conceptualization, Formal analysis, Investigation, Methodology, Writing – original draft, Writing – review & editing. AS: Data curation, Writing – review & editing. CE: Data curation, Writing – review & editing. MN: Investigation, Writing – review & editing. BM: Investigation, Writing – review & editing. PE: Conceptualization, Data curation, Writing – review & editing. J-MB: Conceptualization, Writing – review & editing. EQ: Writing – review & editing. CZ: Conceptualization, Data curation, Investigation, Writing – original draft, Writing – review & editing.

## Supplementary material

Supplementary Figure 1 Masson’s trichrome staining of the bioprinted hydrogel without cell.

Supplementary Figure 2 Immunofluorescence analysis of infected 3D-bioprinted tumor models. 16 days post-infection models were fixed, paraffin embedded and sliced. Consecutive sections were analyzed by immunofluorescent staining against EPCAM (green) **(A)**, and KI67 (purple) and cleaved caspase 3 (green) **(B)** or Vaccinia Virus (Green) (Anti-VV, Creative diagnostics # DMAB4487) **(C)**. As control 3D-bioprinted tumor models mock-treated were analyzed using the same protocol by immunofluorescent staining against EPCAM (green) **(D)**, and KI67 (purple) and cleaved caspase 3 (green) **(E)** or Vaccinia Virus (green) **(F).**

Supplementary Figure 3 Cytotoxicity of oVV on HT29 monolayer. HT29 tumor cells were infected in suspension by oVV-GFP at the indicated MOI. A total of 3 × 10^5 cells/well were plated in 6-well culture dishes in 2 mL of medium supplemented with 10% FCS. Cells were then cultured for 5 days and the viable cells were counted by trypan blue exclusion using a Vi-Cell Cell Counter (Beckman Coulter). All samples were analyzed in triplicate. Results are expressed as percentage of viable cells, 100% corresponding to mock-infected cells. Values are represented as means ± SD of three individual determinations.

Supplementary Figure 4 Quantification of infected cells by flow cytometry. 3D-bioprinted tumor models were infected by 1.10^5 pfu of oVV expressing mCherry. Infection was monitored by fluorescent microscopy **(A, B)**, at 5 days post-infection **(C)** or 28 days post-infection **(D)** models were dissociated by enzymatic digestion and analyzed by flow cytometry to quantified mCherry + cells indicating the oVV infection. Percent of mCherry + cells is indicated in the mCherry+ gate.
