# Supplementary figures and images for: 3D bioprinted CRC model brings to light the replication necessity of an oncolytic vaccinia virus encoding FCU1 gene to exert an efficient anti-tumoral activity
Source: Front Oncol. 2024 Jul 18;14:1384499. doi: 10.3389/fonc.2024.1384499 (PMC11292208; doi:10.3389/fonc.2024.1384499)

### Masson's trichrome staining

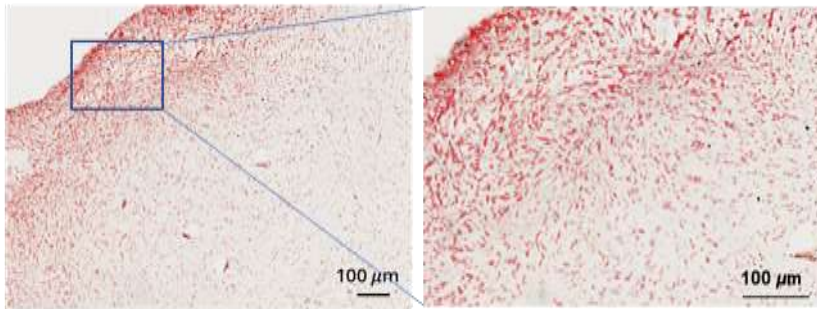

Bio-printed matrix without cells.

Supplement: Supplementary Figure 1 — Masson’s trichrome staining of the bioprinted hydrogel without cell. [file Image_1.pdf]

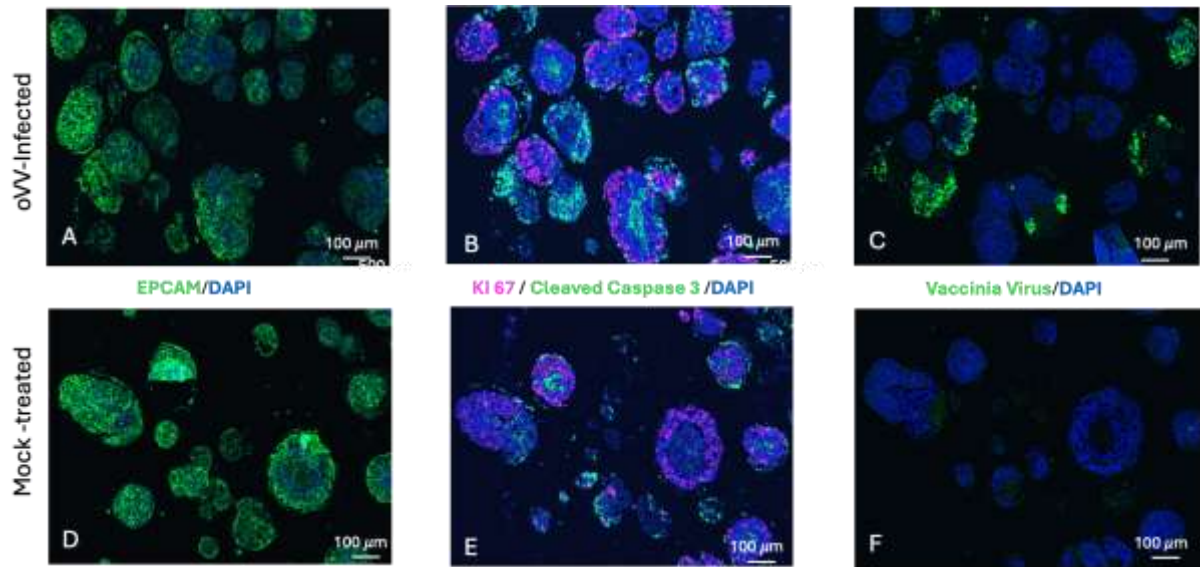

Supplement: Supplementary Figure 2 — Immunofluorescence analysis of infected 3D-bioprinted tumor models. 16 days post-infection models were fixed, paraffin embedded and sliced. Consecutive sections were analyzed by immunofluorescent staining against EPCAM (green) (A), and KI67 (purple) and cleaved caspase 3 (green) (B) or Vaccinia Virus (Green) (Anti-VV, Creative diagnostics # DMAB4487) (C). As control 3D-bioprinted tumor models mock-treated were analyzed using the same protocol by immunofluorescent staining against EPCAM (green) (D), and KI67 (purple) and cleaved caspase 3 (green) (E) or Vaccinia Virus (green) (F). [file Image_2.pdf]

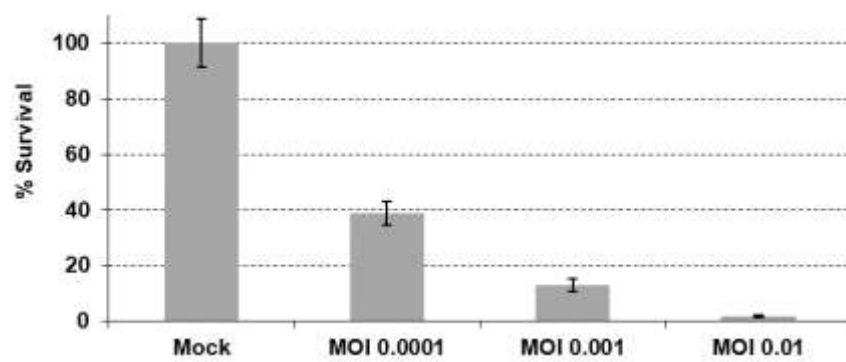

Supplement: Supplementary Figure 3 — Cytotoxicity of oVV on HT29 monolayer. HT29 tumor cells were infected in suspension by oVV-GFP at the indicated MOI. A total of 3 × 10^5 cells/well were plated in 6-well culture dishes in 2 mL of medium supplemented with 10% FCS. Cells were then cultured for 5 days and the viable cells were counted by trypan blue exclusion using a Vi-Cell Cell Counter (Beckman Coulter). All samples were analyzed in triplicate. Results are expressed as percentage of viable cells, 100% corresponding to mock-infected cells. Values are represented as means ± SD of three individual determinations. [file Image_3.pdf]

## D+5 post-infection

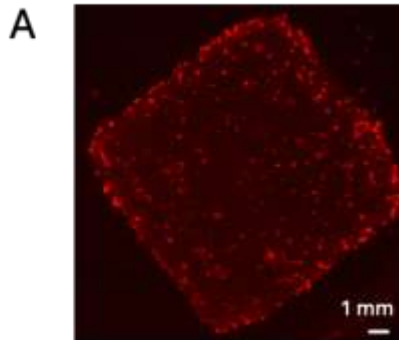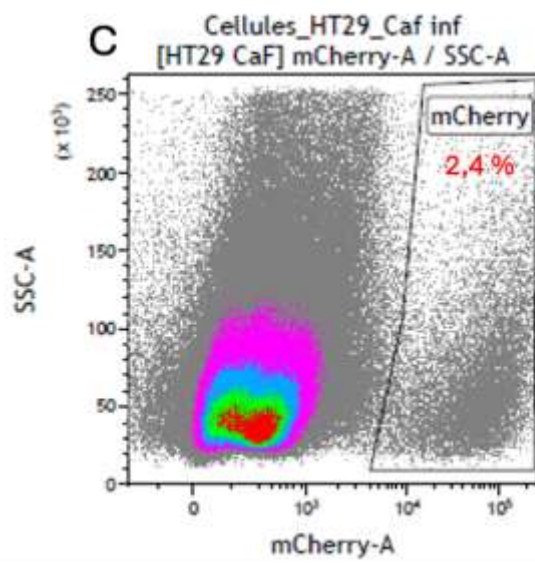

## D+28 post-infection

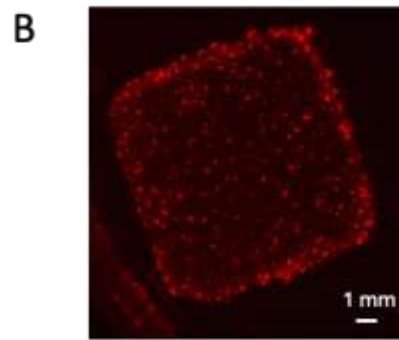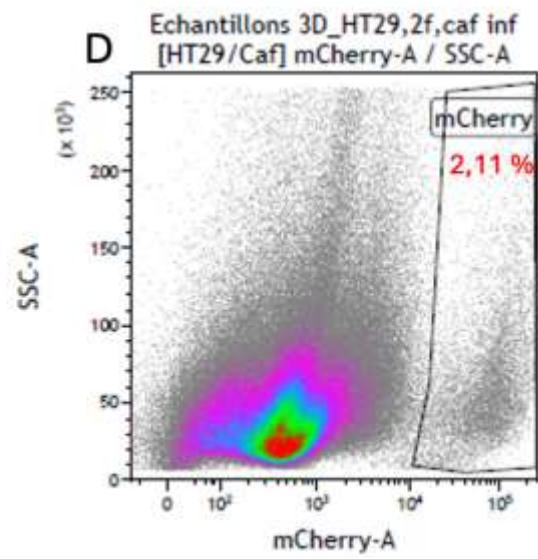

Supplement: Supplementary Figure 4 — Quantification of infected cells by flow cytometry. 3D-bioprinted tumor models were infected by 1.10^5 pfu of oVV expressing mCherry. Infection was monitored by fluorescent microscopy (A, B), at 5 days post-infection (C) or 28 days post-infection (D) models were dissociated by enzymatic digestion and analyzed by flow cytometry to quantified mCherry + cells indicating the oVV infection. Percent of mCherry + cells is indicated in the mCherry+ gate. [file Image_4.pdf]
